# Pterostilbene Simultaneously Induced G0/G1-Phase Arrest and MAPK-Mediated Mitochondrial-Derived Apoptosis in Human Acute Myeloid Leukemia Cell Lines

**DOI:** 10.1371/journal.pone.0105342

**Published:** 2014-08-21

**Authors:** Pei-Ching Hsiao, Ying-Erh Chou, Peng Tan, Wei-Jiunn Lee, Shun-Fa Yang, Jyh-Ming Chow, Hui-Yu Chen, Chien-Huang Lin, Liang-Ming Lee, Ming-Hsien Chien

**Affiliations:** 1 School of Medicine, Chung Shan Medical University, Taichung, Taiwan; 2 Department of Internal Medicine, Chung Shan Medical University Hospital, Taichung, Taiwan; 3 Institute of Medicine, Chung Shan Medical University, Taichung, Taiwan; 4 Graduate Institute of Clinical Medicine, Taipei Medical University, Taipei, Taiwan; 5 Department of Urology, Wan Fang Hospital, Taipei Medical University, Taipei, Taiwan; 6 Department of Medical Research, Chung Shan Medical University Hospital, Taichung, Taiwan; 7 Department of Internal Medicine, Wan Fang Hospital, Taipei Medical University, Taipei, Taiwan; 8 Graduate Institute of Medical Sciences, Taipei Medical University, Taipei, Taiwan; 9 Wan Fang Hospital, Taipei Medical University, Taipei, Taiwan; University of Pecs Medical School, Hungary

## Abstract

**Background:**

Pterostilbene (PTER) is a dimethylated analog of the phenolic phytoalexin, resveratrol, with higher anticancer activity in various tumors. Herein, the molecular mechanisms by which PTER exerts its anticancer effects against acute myeloid leukemia (AML) cells were investigated.

**Methodology and Principal Findings:**

Results showed that PTER suppressed cell proliferation in various AML cell lines. PTER-induced G0/G1-phase arrest occurred when expressions of cyclin D3 and cyclin-dependent kinase (CDK)2/6 were inhibited. PTER-induced cell apoptosis occurred through activation of caspases-8-9/-3, and a mitochondrial membrane permeabilization (MMP)-dependent pathway. Moreover, treatment of HL-60 cells with PTER induced sustained activation of extracellular signal-regulated kinase (ERK)1/2 and c-Jun N-terminal kinase (JNK)1/2, and inhibition of both MAPKs by their specific inhibitors significantly abolished the PTER-induced activation of caspases-8/-9/-3. Of note, PTER-induced cell growth inhibition was only partially reversed by the caspase-3-specific inhibitor, Z-DEVE-FMK, suggesting that this compound may also act through a caspase-independent pathway. Interestingly, we also found that PTER promoted disruption of lysosomal membrane permeabilization (LMP) and release of activated cathepsin B.

**Conclusion:**

Taken together, our results suggest that PTER induced HL-60 cell death via MAPKs-mediated mitochondria apoptosis pathway and loss of LMP might be another cause for cell apoptosis induced by PTER.

## Introduction

Acute myeloid leukemia (AML) is an aggressive malignancy characterized by the rapid growth of abnormal white blood cells (WBCs). AML is primarily treated by chemotherapy, with radiotherapy rarely being applied [1]. Although conventional chemotherapy of AML with either cytarabine or daunorubicin given as a single agent induces complete remission in around 30%∼40% of patients, and combination treatment with both agents induces complete remission in more than 50% of patients [2], only 20%∼30% of patients enjoy long-term disease-free survival [2], and these chemotherapeutic drugs can also affect normal cells causing unpleasant side effects such as anemia, bleeding, and infection. Thus, there is a need for new agents to treat AML.

Over the years, stilbene-based compounds have attracted the attention of many researchers due to their wide range of biological activities. One of the most relevant and extensively studied stilbenes is resveratrol (RESV), a phytoalexin present in grapes and other foods, which is capable of acting as a cancer chemopreventive agent [3], [4]. Indeed, several in vitro and in vivo studies showed that RESV has powerful growth-inhibitory and apoptosis-inducing effects on various solid tumor cells, including colon, breast, prostate, cervical, and pancreatic cancers [5]–[9]. As to the effects of RESV on non-solid tumors, several studies also indicated that RESV is particularly active in continuous leukemic cells, and it is capable of suppressing the colony-forming cell proliferation of fresh AML marrow cells from patients with AML [10], [11]. Despite its promising properties, RESV’s rapid metabolism and low bioavailability have precluded its advancement to clinical use [12]. Limitations of RESV prompted our interest in natural and synthetic analogues with improved pharmacokinetics and superior pharmacological potencies that hold greater potential as natural anticancer drugs.

**Figure 1:**
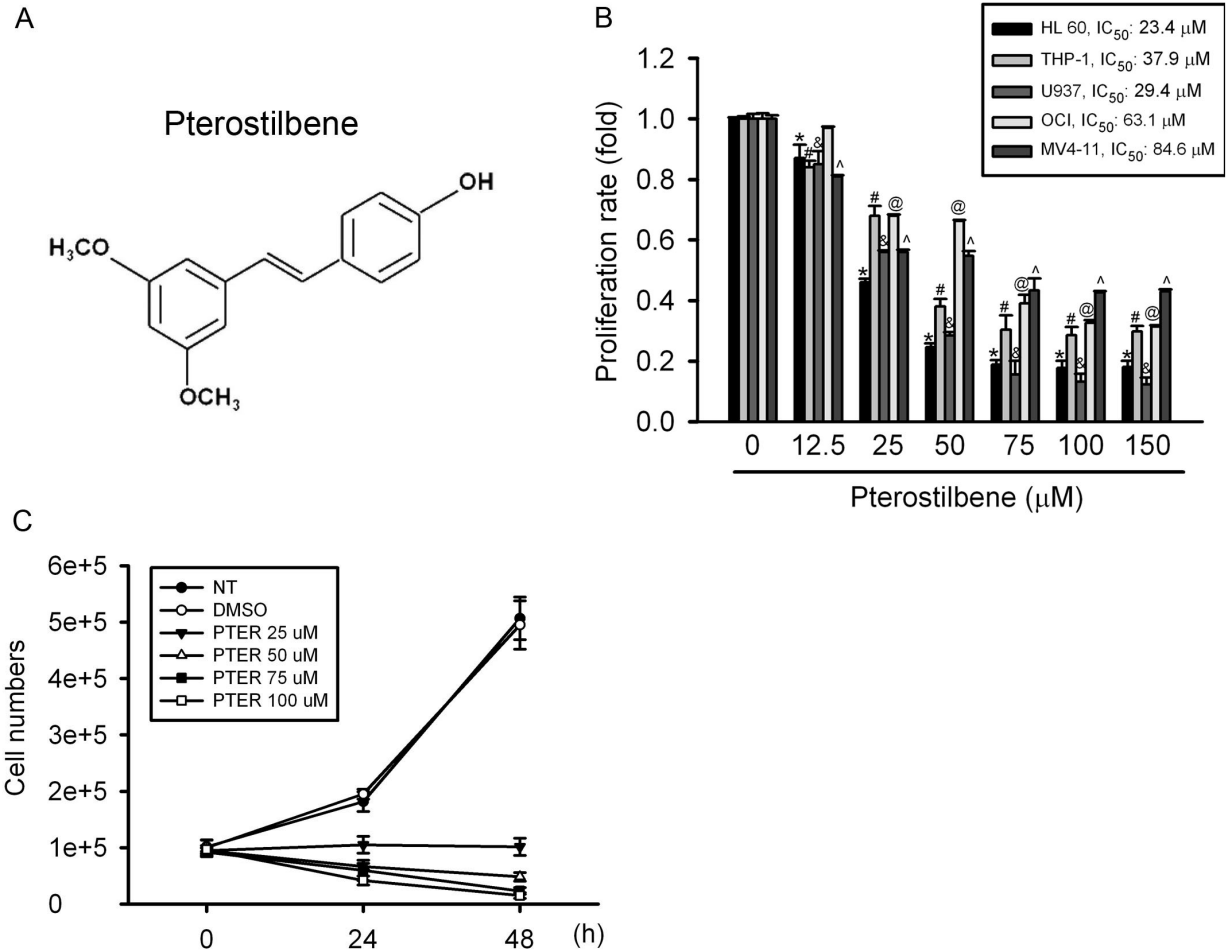
Effect of pterostilbene (PTER) on the cell proliferation of acute myeoloid leukemia (AML) cell lines. (A) The chemical structure of PTER. (B) Five AML cell lines were treated with the vehicle (DMSO) or PTER (12.5∼150 µM) in serum-containing medium for 24 h. Cell proliferation was determined by an MTS assay. Results are expressed as multiples of cell proliferation rate. Values represent the mean ± SE of 3 independent experiments. *^, #, &, @, ∧^ *p*<0.05, compared to the vehicle groups. (C) HL-60 cells were treated with different concentrations of PTER (0∼150 µM) for 24 and 48 h and analyzed by a trypan blue exclusion assay. Quantitative assessment of the mean number of cells is expressed as the mean ± SE.

Pterostilbene (PTER) (trans-3,5-dimethoxy-4-hydroxystilbene, Figure. 1A), a natural dimethylated analog of RESV, was proposed to have similar properties as RESV including anticancer, anti-inflammation, antioxidant, apoptosis, antiproliferation, and analgesic potential [13]. Under most circumstances, PTER is either equally or significantly more potent than RESV [14], [15]. Most importantly, following equimolar oral dosing in rats, plasma levels of PTER were markedly greater than those of RESV [16]. The greater bioavailability of PTER indicates that PTER could potentially be developed for clinical applications. Indeed, many studies confirmed that PTER exerts antiproliferative and proapoptotic effects in both solid (e.g., lung, gastric, prostate, colon, and breast cancers) [15], [17]–[20] and non-solid tumors (e.g., chronic myelogenous leukemia and lymphoblastic leukemia) [21], [22]. However, the mechanisms of PTER activity in cancer cell lines, especially against leukemic cells, have not been fully elucidated.

In this study, we examined the antitumor activities of PTER in five different human AML cell types. Furthermore, we explored the effects of PTER on the mitochondrial and lysosomal apoptotic pathways and cell cycle-related proteins in AML cells.

## Materials and Methods

### Materials

PTER of 98% purity was purchased from Enzo Life Sciences (Lausen, Switzerland). A 100 mM stock solution of PTER was made in dimethyl sulfoxide (DMSO) (Sigma, St. Louis, MO) and stored at −20°C. The final concentration of DMSO for all treatments was <0.5%. Antibodies, specifically of cleaved caspase-3, caspase-8, caspase-9, poly(ADP-ribose) polymerase (PARP), heat shock protein 70 (HSP70), p-extracellular signal-regulated kinase (ERK)1/2, p-p38, p-c-Jun N-terminal kinase (JNK), ERK1/2, p38, JNK1/2, CDK 2, cathepsin B, C23, and β-actin (for the Western blot analysis), were purchased from Santa Cruz Biotechnology (Santa Cruz, CA). Antibodies against cyclin-dependent kinase (CDK)6, p21 Cip1, p27 Kip1, p15 INK4B, and cyclin D3 were purchased from Cell Signaling Technology (Danvers, MA). Anti-cyclin A2 and anti-cyclin E2 antibodies were purchased from Epitomic (Burlingame, CA). 4′-6-Diamidino-2-phenylindole (DAPI) was purchased from Sigma. The p38 mitogen-activated protein kinase (MAPK) inhibitor, SB202190, the ERK1/2 inhibitor, U0126, and the JNK1/2 inhibitors, SP600125 and JNK-IN-8, were purchased from Calbiochem (San Diego, CA). The caspase-3 inhibitor, Z-DEVE-FMK, was purchased from BioVision (Mountain View, CA). Unless otherwise specified, other chemicals used in this study were purchased from Sigma.

### Cell Culture

The human MV4-11 HL-60, U937, and THP-1 AML cell lines were purchased from the American Type Culture Collection (Manassas, VA), while the OCI-AML3 cell line was purchased from DSMZ (Braunschweig, Germany). All cell lines were cultured in RPMI 1640 medium supplemented with 10% heat-inactivated fetal bovine serum (FBS; Gibco, Grand Island, NY), 2 mM L-glutamine, 100 U/ml penicillin, and 100 µg/ml streptomycin.

### In Vitro Cytotoxicity Assay

AML cells (OCI-AML3, MV4-11, HL-60, U937, and THP-1) were plated in 96-well microtiter plates and treated with various concentrations (0, 12.5, 25, 50, 75, 100, and 150 µM) of PTER for 24 h, and cell viabilities were assessed using an MTS (Promega, Madison, WI) assay. The absorbance (A) was read at 490 nm using an enzyme-linked immunosorbent assay (ELISA) reader (MQX200; Bio-Tek Instruments, Winooski, VT). The cell viability rate (multiple) was determined by the formula: A_490, PTER_/A_490, vehicle_.

### Cell Proliferation Assay

The proliferation of AML cells was measured by directly counting cells with a hemocytometer. Briefly, cells were seeded at a density of 10^5^ cells/well in a 12-well culture plate, grown in RPMI containing 10% FBS and then treated with 0.5% DMSO without (control) or with various concentrations of PTER. Medium without or with PTER was changed daily until cells were counted.

### Flow Cytometric Analysis

HL-60 cells (2×10^6^/ml) were treated with 0.5% DMSO or PTER (0∼100 µM) for 24 h. At the end of incubation, cells were collected and fixed with 70% ethanol. Cells were stained with propidium iodide (PI) buffer (4 µg/ml PI, 1% Triton X-100, and 0.5 mg/ml RNase A in phosphate-buffered saline (PBS)) for 30 min in the dark at room temperature and then filtered through a 40-µm nylon filter (Falcon, San Jose, CA). The cell cycle distribution was analyzed for 10,000 collected cells by a FACS Vantage flow cytometer that uses the Cellquest acquisition and analysis program (Becton-Dickinson FACS Calibur, San Jose, CA). The proportion of nuclei in each phase of the cell cycle was determined, and apoptotic cells with hypodiploid DNA content were detected in the sub-G_1_ region. All results were obtained from three independent experiments.

### Annexin-V/PI Staining Assay

Apoptosis-mediated cell death of tumor cells was examined using a double-staining method with an FITC-labeled Annexin-V/PI Apoptosis Detection kit (BD Biosciences, San Jose, CA). For PI and Annexin-V double-staining, cells were suspended in 100 µl of binding buffer (10 mM HEPES/NaOH, 140 mM NaCl, and 2.5 mM CaCl_2_ at pH 7.4) and stained with 5 µl of FITC-conjugated Annexin-V and 5 µl of PI (50 µg/ml) for 30 min at room temperature in the dark, and then 400 µl of binding buffer was added. Apoptotic cells were analyzed via flow cytometry, with a FACScan system flow cytometer. Data were acquired and analyzed in a Becton-Dickinson FACS Calibur flow cytometer using Cell Quest software.

### DAPI Staining

HL-60 cells were treated with 100 µM PTER for 24 h and were then seeded on a slide via cytospinning. Apoptotic morphological changes were assessed using DAPI staining, as previously described [23].

### Western Blot Analysis

Total cell lysates and cytosolic protein extraction were prepared as previously described [24]. Equal amounts of protein extracts (2∼0 µ 30 g) were subjected to 10% or 12% sodium dodecylsulfate polyacrylamide gel electrophoresis (SDS-PAGE) and blotted onto polyvinylidene fluoride membranes (Millipore, Belford, MA). After blocking, membranes were incubated with primary antibodies for CDK 2, CDK6, cyclin A2, cyclin D3, cyclin E2, p15 INK4B, p21 Cip1, p27 Kip1, caspases-9, -3, and -8, PARP, HSP70, ERK1/2, p-ERK1/2, p38, p-p38, JNK1/2, p-JNK1/2, cathepsin B, and β-actin. Blots were then incubated with a horseradish peroxidase (HRP)-conjugated anti-mouse or anti-rabbit antibody. Signals were detected via enhanced chemiluminescence using Immobilon Western HRP Substrate (Millipore, Billerica, MA).

### Mitochondrial Membrane Potential (MMP) Measurement

Breakdown of the mitochondrial membrane potential was assessed by FACS analyses or epifluorescent microscopy (Zeiss Axioplan) using JC-1 (5,5′,6,6′-tetra-chloro-1,1′,3,3′-tetra-ethylbenzimidazol-carbocyanine iodide), which allows detection of changes in the MMP. For this purpose, a Mitochondrial Membrane Potential Detection Kit (Immunochemistry, Bloomington, MN) was used, as described in the manufacturer’s instructions. HL60 cells at 10^6^ were treated for 24 h with PTER. After PBS washing, cells were incubated for 15 min in freshly prepared JC-1 solution (10 µg/ml in culture medium) at 37°C. Spare dye was removed by PBS washing, and cell-associated fluorescence was measured with FACS and fluorescent microscopy, respectively.

### Lysosome Membrane Permeability (LMP) Analysis

Acridine orange (AO) was used to determine changes in LMP [25]. In brief, 10^6^ HL60 cells were treated for 12 h with PTER. AO was then added (at a final concentration of 5 µg/ml), and cells were incubated for another 15 min. Cells were washed twice with PBS and examined with an epifluorescence microscope (Zeiss Axioplan). The excitation and emission wavelengths of red fluorescence were 555 and 617 nm and those of green fluorescence were 490 and 528 nm, respectively. A previous study demonstrated that AO concentrated in lysosomes emits granular red fluorescence, whereas AO in the cytosol emits diffuse green fluorescence. Following a change in lysosome permeability, increased diffuse cytosolic green fluorescence can be observed, indicating a relocation of AO from the lysosomes to the cytosol [25].

### Measurement of ROS Production

HL-60 cells were treated with PTER for the indicated time; then ROS production was measured by staining with ROS probes (5 µM DCFDA) in RPMI 1640 medium for 30 min. After being washed with PBS or medium, the ROS production of DCFDA-preloaded cells was measured by FACS.

### Statistical Analysis

Values are shown as the mean ± standard error (SE). Statistical analyses were performed using the Statistical Package for Social Science software, vers. 16 (SPSS, Chicago, IL). Data comparisons between two groups were performed with Student’s *t*-test. A one-way analysis of variance (ANOVA) followed by Tukey’s post-hoc test was used when more than three groups were analyzed. Differences were considered significant at the 95% confidence level when *p*<0.05.

## Results

### Effect of PTER on Cell Proliferation of AML Cell Lines

The chemical structure of PTER is shown in Figure 1A. To determine the in vitro efficacy of PTER in AML cells, we treated several AML cell lines with PTER. The cytotoxic effects of PTER were examined in five AML cell lines which represent different French-American-British (FAB) types (M2: HL-60; M4: OCI-AML3; and M5: MV4-11, U937, and THP-1). As shown in Figure 1B, after treatment for 24 h, PTER significantly reduced cell proliferation in a concentration-dependent manner, and 50% growth inhibitory concentration (IC_50_) values were around 20∼80 µM for the five AML cell lines. In these five AML cell lines, HL-60 cells were the most sensitive to PTER treatment (IC_50_: 23.4 M). We further studied the antiproliferative activity of PTER against HL-60 cells by counting cells. As illustrated in Figure 1C, PTER time- and concentration-dependently decreased the number of cultured HL-60 cells. These results indicated that PTER can potently inhibit the proliferation of different AML FAB types.

### PTER Simultaneously Induces HL-60 Cell Apoptosis and Cell Cycle Arrest

**Figure 2:**
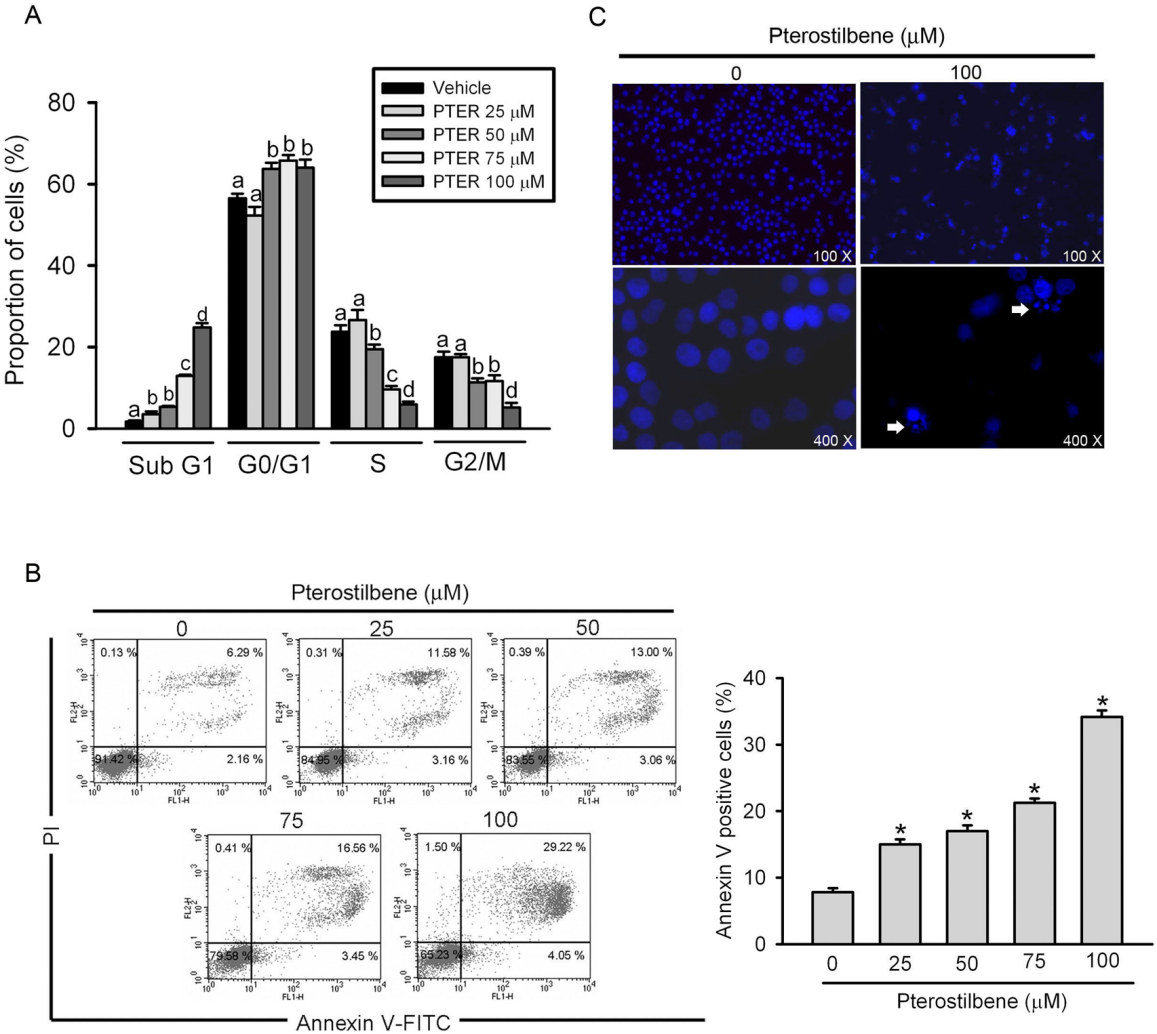
Effect of pterostilbene (PTER) on HL-60 cell-cycle regulation and apoptosis. (A) HL-60 cells were treated with different concentrations of PTER (0∼100 µM) for 24 h, and flow cytometry was used to detect the cell cycle phase distribution and cell death in the sub-G_1_ phase. Data are presented as the mean ± SE of three independent experiments. Results were analyzed using one-way ANOVA with Tukey’s post hoc tests at 95% confidence intervals. Different letters represent significantly different, *p*<0.05. (B) Quantitative analysis of cell apoptosis by Annexin-V and propidium iodide (PI) double-staining flow cytometry. Values represent the mean ± SE of three independent experiments. *p<0.05, compared to the vehicle group. (C) HL-60 cells were treated with 100 µM PTER for 24 h and analyzed by fluorescence microscopy after DAPI staining. White arrows indicate apoptotic HL-60 cells.

Physiological cell death is characterized by apoptotic morphology, including chromatin condensation, membrane blebbing, internucleosomal degradation of DNA, and apoptotic body formation. To investigate the mode of cell death induced by PTER, HL-60 cells were treated with PTER (0∼100 µM) for 24 h. It was shown that PTER induced concentration-dependent increases of the sub-G_1_ population (Figure 2A). Similarly, as shown in Figure 2B, we assessed the translocation of phosphatidylserine (PS) using Annexin-V and PI double-staining. Apoptotic cells (PI-negative/Annexin-V-positive and PI-positive/Annexin-V-positive) increased from 8.45% to 14.74%, 16.06%, 20.01%, and 33.27% after respectively treating HL-60 cells with 25, 50, 75, and 100 µM PTER. Actually, we can’t exclude the possibility that the necrotic cells also involved in PI-positive/Annexin-V-positive populations. Next, the effect of PTER on cell morphology was further examined using fluorescence microscopy. Cells treated with 100 µM PTER for 24 h demonstrated morphologies characteristic of apoptosis, such as chromatin condensation and formation of apoptotic bodies (Figure 2C, arrows). These results are all hallmarks of apoptotic cell death and demonstrated the ability of PTER to induce apoptosis in HL-60 cells. Moreover, the similar effect of PTER on the translocation of PS was also observed in another AML cell line, U937 (Figure S1A in File S1). In addition to apoptosis induction by PTER, we further found another mechanism responsible for the growth inhibition induced by PTER. As shown in Figure 2A, more cells were arrested at the G_0_/G_1_ phase of the cell-cycle after PTER (50∼100 µM) exposure for 24 h (Figure 2A).

### Effect of PTER on Alterations of Cell-cycle Regulatory Proteins

**Figure 3:**
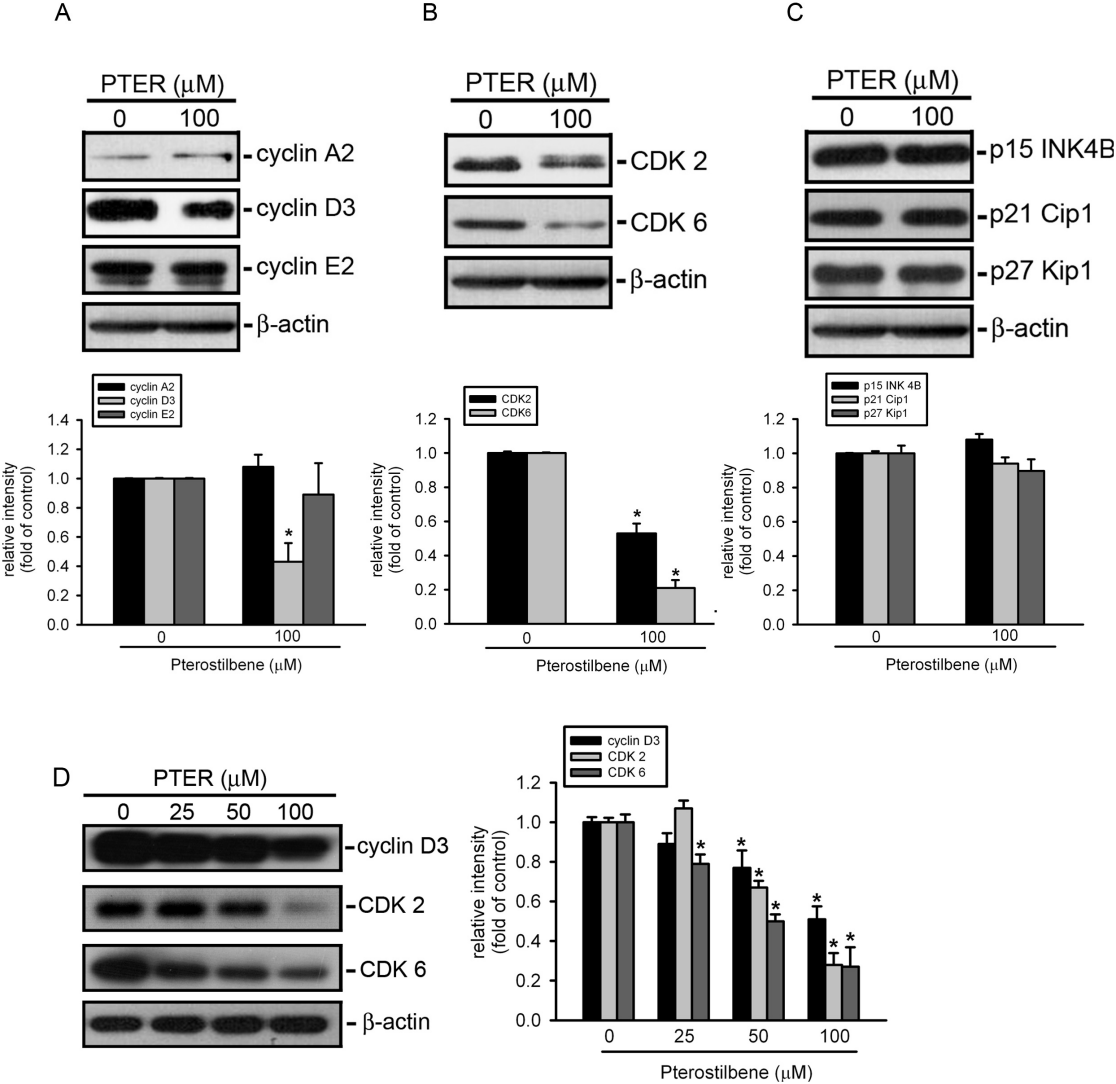
Effect of pterostilbene (PTER) on alterations of cell-cycle regulatory proteins in HL-60 cells. Proteins were extracted from cultured HL-60 cells at 24 h after PTER treatment and probed with proper dilutions of specific antibodies. (A and B) PTER at a concentration of 100 µM induced significant decreases in protein levels of cyclin D3, CDK2, and CDK6. Upper panels: Representative results of cyclins and cyclin-dependent kinase (CDK) protein levels as determined by a Western blot analysis. Lower panels: Quantitative results of cyclin and CDK protein levels, which were adjusted to the β-actin protein level and expressed as multiples of induction beyond its own control. Values are presented as the mean ± SE of three independent experiments. *p<0.05, compared to the vehicle control group. (C) There were no significant differences in protein levels of p15 INK4B, p21 Cip1, or p27 Kip1 between control and PTER-treated HL-60 cells. Upper panel: Representative results of p15, p21, and p27 protein levels as determined by a Western blot analysis. Lower panel: Quantitative results of p15, p21, and p27 protein levels, which were adjusted with the β-actin protein level and expressed as multiples of induction beyond its own control. (D) Cyclin D3, CDK2, and CDK6 peotein expression were downregulated in a concentration-dependent fashion after PTER treatment in HL-60 cells. Left panel: Representative results of cyclin D3, CDK2, and CDK6 protein levels as determined by a Western blot analysis. Right panel: Quantitative results of cyclin D3, CDK2, and CDK6 protein levels, which were adjusted with the β-actin protein level and expressed as multiples of induction beyond its own control. *p<0.05, compared to the vehicle control group.

To investigate the molecular mechanisms underlying PTER-induced G_0_/G_1_ arrest, cells were cultured in media supplemented with 10% FBS and 0.5% DMSO without or with PTER (100 µM), and at 24 h, they were harvested for protein extraction and a Western blot analysis. As shown in the Figure 3A and 3B, levels of cyclin D3, CDK2, and CDK6 proteins had significantly decreased in PTER-treated HL-60 cells. Because CDK activity can be controlled by a group of cyclin-dependent kinase inhibitors (CKIs), we examined protein levels of p15 INK4B, p21Cip1, and p27Kip1, three known CKIs, in PTER-treated HL-60 cells. However, levels of p15 INK4B, p21Cip1, and p27Kip1 proteins exhibited no significant change in PTER-treated HL-60 cells compared to DMSO-treated cells (Figure 3C). Moreover, expression of cyclin D3, CDK2, and CDK6 proteins can also be suppressed significantly by lower concentration of PTER (50 µM) in HL-60 cells (Figure 3D).

### PTER Induces Caspase Activation and Changes in the MMP in HL-60 Cells

**Figure 4:**
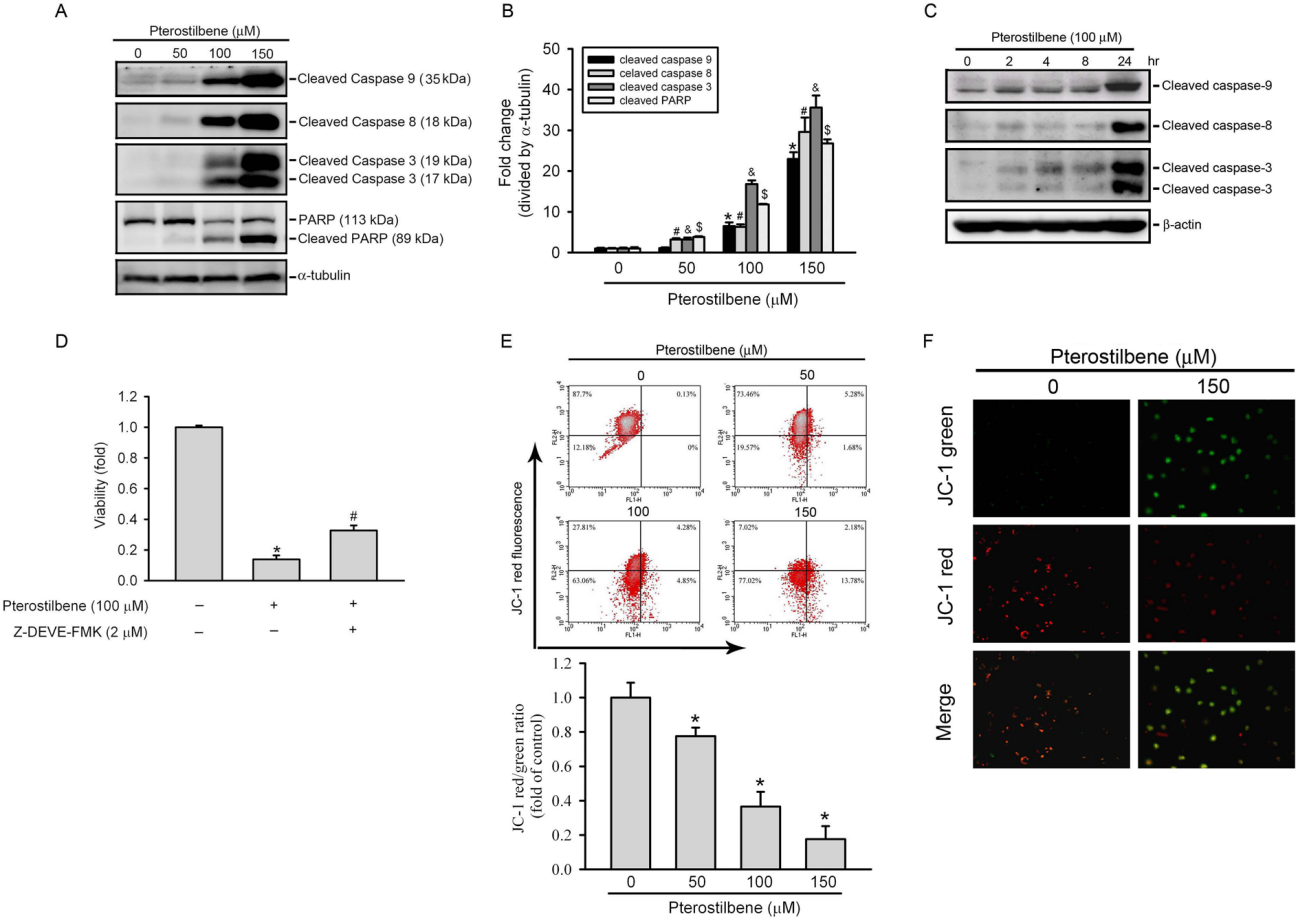
Effect of pterostilbene (PTER) on caspase activation and mitochondrial membrane permeability (MMP) in HL-60 cells. (A) Expression levels of cleaved caspases-3, -8, and -9, and poly (ADP-ribose polymerase (PARP) were assessed by a Western blot analysis after treatment with various concentrations of PTER (0∼150 µM) for 24 h. (B) Quantitative results of cleaved caspase-3, -8, and -9, and PARP protein levels, which were adjusted to the α-tubulin protein level and expressed as multiples of induction beyond each respective control. Values are presented as the mean ± SE of three independent experiments. *^, #, &, $^ *p*<0.05, compared to the vehicle control groups. (C) Activated caspase-9, -8, and -3 protein expression were upregulated in a time-dependent fashion after PTER 100 µM) treatment, peaking at 24 h in HL-60 cells. (D) Effect of a caspase-3 inhibitor on PTER-induced cell death. Cells were treated with 100 µM PTER for 24 h in the presence or absence of 2 µM Z-DEVE-FMK. Cell proliferation was determined by an MTS assay. Data are presented as the mean ± SE of three independent experiments performed in triplicate. *p<0.05, control vs. PTER; ^#^ *p*<0.05, PTER vs. Z-DEVE-FMK plus PTER. (E and F) Loss of the MMP after 24 h of treatment with PTER as determined by FACS and immunofluorescence analyses of JC-1 staining. (E) From the FACS analysis, increased percentages of green fluorescent apoptotic (FL 1) populations of HL-60 at the indicated drug concentrations (cells in the lower right field) are indicated (upper panel). The red-to-green fluorescence ratio indicates functional mitochondria with membrane potential. Data are presented as the mean ± SE of three independent experiments performed in triplicate. *p<0.05, control vs. PTER (F) Immunofluorescence analysis showed that green-fluorescent monomeric form increases in HL-60 cells after treatment with 100 µM PTER for 24 h. Original magnification, 200×.

The apoptotic process is executed by a member of the highly conserved caspases, and modulation of the mechanisms of caspase activation and suppression is a critical molecular target in chemoprevention, since these processes lead to apoptosis [26]. To identify the mechanisms underlying PTER-induced apoptosis in HL-60 cells, activation of caspases-8, -9, and -3 and cleavage of PARP were detected. Figure 4A shows that exposure of HL-60 cells to PTER (0∼150 µM for 24 h) caused concentration-dependent increases in the activation of caspases-8, -9, and -3, and cleaved PARP. PTER treatment at 50, 100, and 150 µM for 24 h significantly increased expression levels of cleaved PARP by 3.81, 11.83-, and 26.98-fold, respectively, compared to the control (Figure 4B). Moreover, treatment of HL-60 cells with PTER (100 µM) also resulted in a time-dependent increase in activated caspases-8, -9, and -3, with a maximal effect at 24 h (Figure 4C). In addition, the PTER (100 µM)-mediated activation of caspases-8, -9, and -3 was also observed in U937 cells (Figure S1B in File S1). In order to further explore the significance of caspase activation in PTER-induced apoptosis, a specific inhibitor of executioner caspase-3, Z-DEVE-FMK, was used to suppress the effect of PTER. Pretreatment with Z-DEVE-FMK partially attenuated PTER-induced inhibition of proliferation (Figure 4D), suggesting that PTER-mediated anti-proliferative effect partially occurs through a caspase-dependent apoptosis pathway.

To further detect whether PTER-induced apoptotic cell death involves a mitochondrial pathway, the fluorescent cationic dye, JC-1, was used to detect the mitochondrial permeability transition. Collapse of the MMP is an early step in the induction of apoptosis by the intrinsic pathway [27]. In healthy, non-apoptotic cells, the dye accumulates and aggregates within mitochondria, resulting in bright-red staining. In apoptotic cells, due to the collapse of the membrane potential, JC-1 cannot accumulate within mitochondria and remains in the cytoplasm in its green-fluorescent monomeric form. FACS and immunofluorescence analyses of JC-1-stained HL60 cells treated with PTER for 24 h are shown in Figure 4E and 4F. PTER induced concentration-dependent collapse of the MMP (Figure 4E). Moreover, our results showed that treatment of cells with PTER in earlier stages (4 and 6 h) also can induce the changes of mitochondrial membrane potential (Figure S2 in File S1).

### ERK1/2 and JNK1/2 Are Essential for Caspase-8, -9, and -3 Activation Induced by PTER

**Figure 5:**
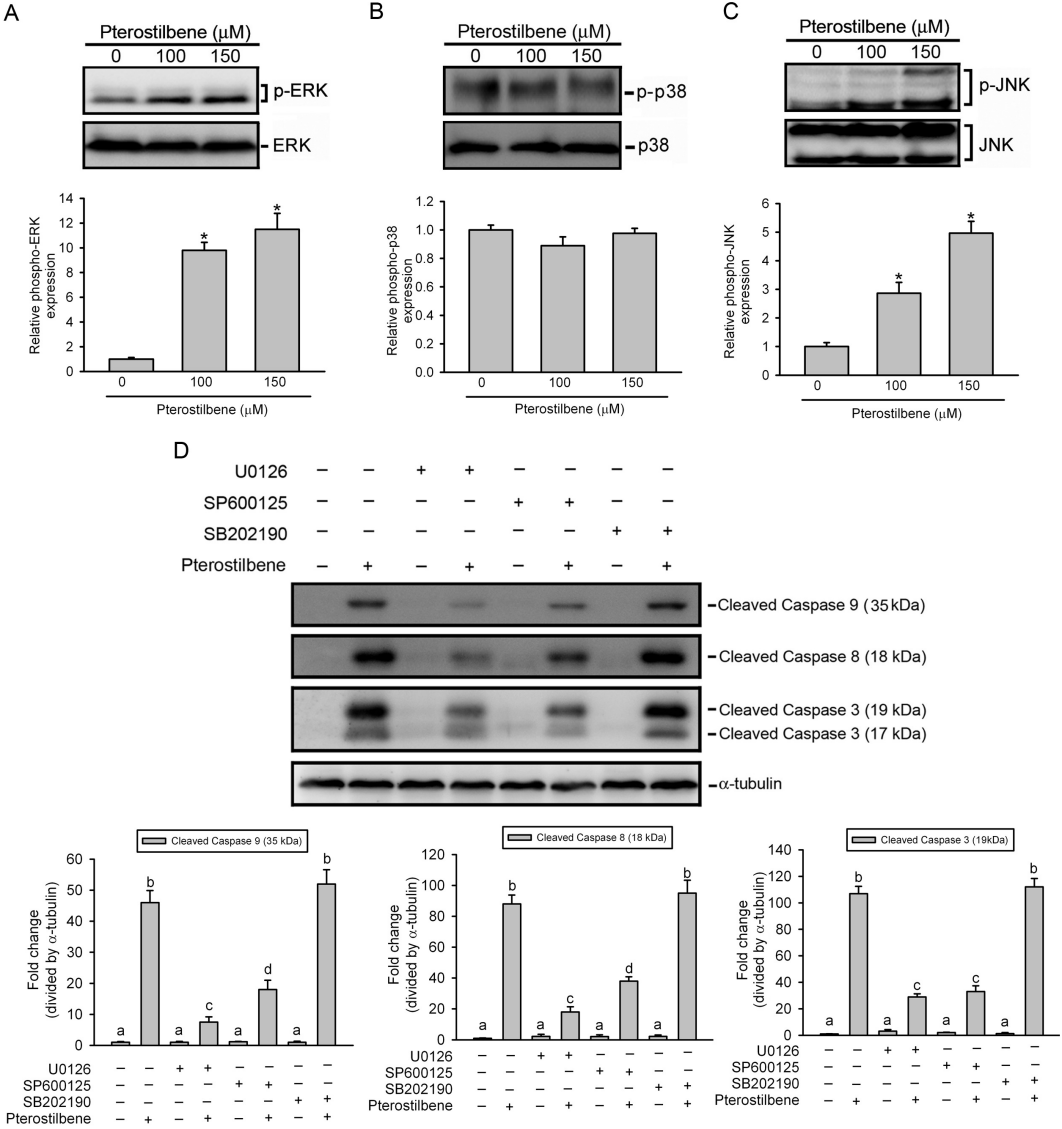
Role of mitogen-activated protein kinase (MAPKs) in pterostilbene (PTER)-induced activation of caspases-8,-9, and -3. (A–C, upper panel) Phosphorylation levels of extracellular signal-regulated kinase (ERK)1/2, p38, and c-Jun N-terminal kinase (JNK)1/2 were assessed by a Western blot analysis after treatment with various concentrations of PTER (0∼150 µM M) for 24 h. (A–C, lower panel) Quantitative results of phopho-ERK1/2, p38, and JNK1/2 protein levels, which were adjusted with the total ERK1/2, p38, and JNK1/2 protein levels and expressed as multiples of induction beyond each respective control. Values represent the mean ± SE of three independent experiments. *p<0.05, compared to the vehicle control group. (D, upper panel) HL-60 cells were pretreated with or without 20 µM U0126, SP600125, or SB202190 for 1 h followed by PTER (100 µM) treatment for an additional 24 h. Expression levels of cleaved caspase-3, -8, and -9 were determined by a Western blot analysis. (D, lower panel) Quantitative results of cleaved caspase-3, -8, and -9 protein levels, which were adjusted to the α-tubulin protein level and expressed as multiples of induction beyond each respective control. Values represent the mean ± SE of three independent experiments. Data were analyzed using a one-way ANOVA with Tukey’s post-hoc tests at 95% confidence intervals; different letters represent different levels of significance. Different letters represent significantly different, *p*<0.05.

Previous studies showed that the MAPK signaling pathway plays an important role in the action of chemotherapeutic drugs [28]. Therefore, we determined whether MAPKs were activated in PTER-treated HL-60 cells, and found that PTER induced activation of JNK1/2 and ERK1/2, but not p38 MAPK in dose-dependent manners (Figure 5A–C). Next, we further investigated relationships among PTER-induced activation of caspases-8, -9, and -3, and MAPKs. HL-60 cells were pretreated with 20 µM U0126 (an ERK inhibitor), SP600125 (a JNK inhibitor), or SB202190 (a p38 inhibitor) for 1 h, treated with 100 µM PTER for another 24 h, and then analyzed by Western blotting. The concentrations of U0126 and SP600125 we used here showed significant inhibitory effects on PTER-induced ERK1/2 and JNK activation (Figure S3 in File S1). As shown in Figure 5D, both U0126 and SP600125 significantly attenuated PTER-induced caspase-8, -9, and -3 activation. To consider the specificity of SP600125, we further used another selective inhibitor of JNK, JNK-IN-8, and found pretreatment of JNK-IN-8 (1 µM) with HL-60 cells also suppressed PTER-induced caspase-8, -9, and -3 activation obviously (Figure S4 in File S1). Moreover, we also found that JNK1/2 and ERK1/2 activation occurred at earlier time than caspases-8, -9, and -3 activation after PTER treatment (Figure 4C and Figure S5 in File S1). These findings suggest that activation of ERK1/2 and JNK1/2 might play critical roles in PTER-mediated caspases activation in HL-60 cells.

### PTER Induces Lysosomal Membrane Alterations

**Figure 6:**
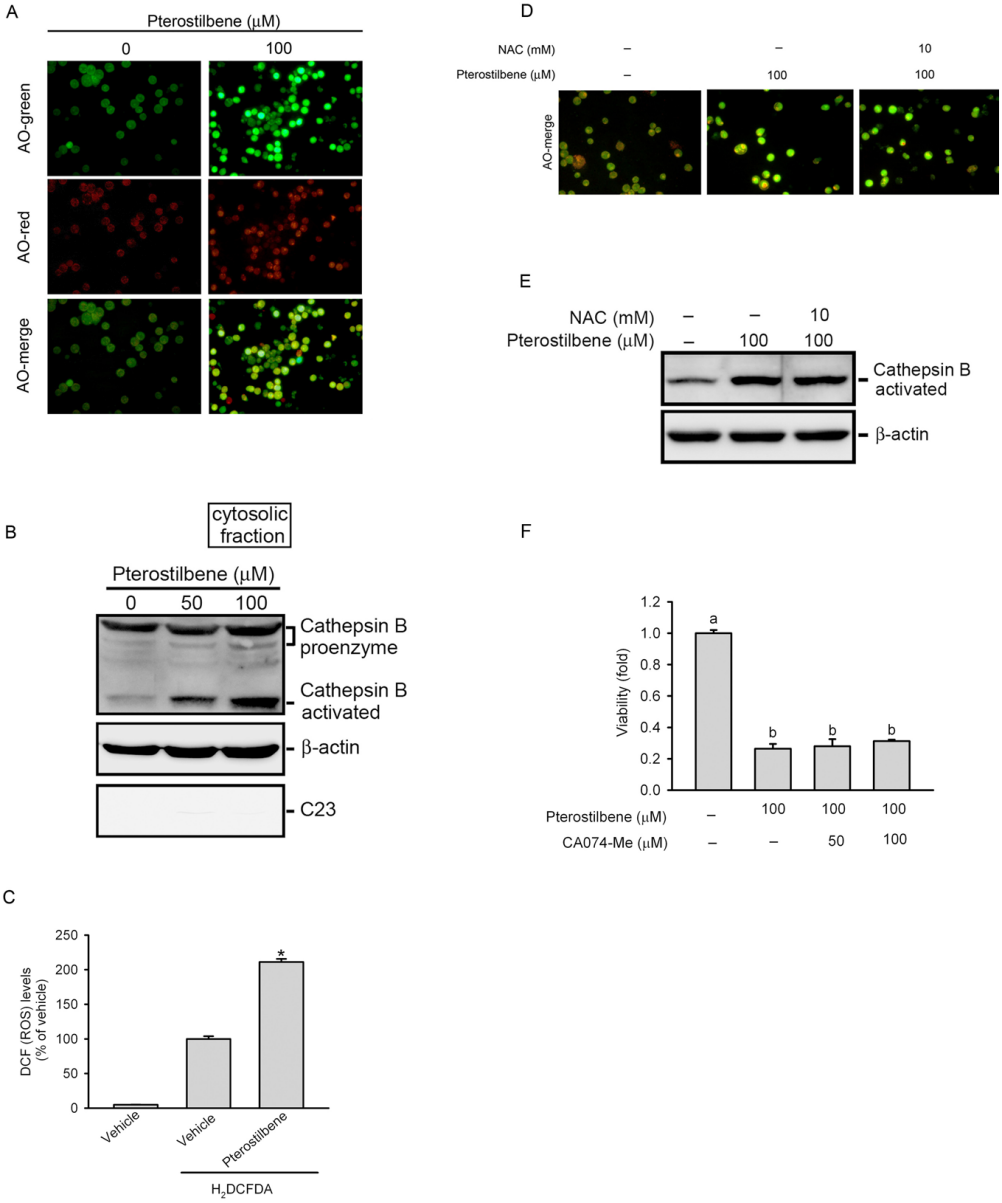
Effect of pterostilbene (PTER) on lysosomal membrane alterations in HL-60 cells. (A) PTER enhanced lysosome permeability. HL-60 cells were treated with 100 µM PTER for 12 h, then stained with acridine orange (5 µg/ml) for 15 min, and examined under a fluorescence microscope (×200 magnification). Representative images of three independent experiments are shown. (B) PTER concentration-dependently induced translocation of cathepsin B from lysosomes to the cytosol. Cytosolic cathepsin B levels were assessed by a Western blot analysis after treatment with various concentrations of PTER (0∼100 µM) for 24 h. Protein levels of both the 37-kDa pro-cathepsin and 25-kDa activated cathepsin B are shown. β-actin and C23 were respectively used as positive and negative cytosolic internal controls. (C) HL-60 cells were treated with 100 µM PTER for 1 h and stained with H_2_DCFDA; then total ROS level was analyzed by FACS, and data are presented as the mean multiples of increase in fluorescence compared to the control ± SE. *p<0.05, compared to the control. (D and E) The reactive oxygen species (ROS) scavenger, N-acetyl cysteine (NAC; 10 mM) was added 1 h prior to the addition of 100 µM PTER. Lysosome permeability (D) and cathepsin B cytosolic translocation (E) were analyzed 24 h later. (F) The cathepsin B inhibitor, CA074-Me (50 and 100 µM), was added 1 h prior to the addition of 100 µM PTER. Cell proliferation was determined by an MTS assay. Values are presented as the mean ± SE of three independent experiments. Data were analyzed using a one-way ANOVA with Tukey’s post-hoc tests at 95% confidence intervals; Different letters represent significantly different, *p*<0.05.

Notably, our results showed that the caspase-3 inhibitor, Z-DEVE-FMK, only partially reversed PTER-induced cell death (Figure 3C). This result suggests that caspase-independent mechanisms might also play a role in PTER-induced cytotoxicity. Lysosomal leakage was described as an inducer of cell death [29], and lysosomal destabilization was shown to be a common consequence of microtubule-targeting drugs [30]. Thus, to further elucidate the mechanisms underlying PTER-induced cancer cell death, we studied its possible effects on lysosomes. Lysosomal permeability was examined using AO staining. AO is a metachromatic fluorescent cationic dye that emits red light at high concentrations inside lysosomes, and green light in the cytosol. Following a change in lysosomal permeability, increased diffuse cytosolic green fluorescence can be observed, indicating a relocation of AO from lysosomes to the cytosol [25]. As shown in Figure 6A, dramatic increases in the diffuse cytosolic green fluorescence were observed in PTER-treated HL-60 cells. Next, we analyzed the release of lysosomal cathepsin B to the cytosol as a marker of lysosomal membrane permeabilization (LMP). Remarkably, PTER caused a concentration-dependent increase in activated cathepsin B in the cytosol in HL-60 cells after 24 h of PTER treatment (Figure 6B). These results indicated that PTER can induce changes in lysosomal permeability. Several studies reported that reactive oxygen species (ROS) production induces permeabilization of lysosomes [31]. To determine whether ROS are induced by PTER in HL-60 cells, cellular ROS were monitored with the redox-sensitive dyes, H_2_DCFDA. Our results showed that in compared to the control group, treatment of cells with 100 µM PTER significantly increased ROS production (Figure 6C). To investigate the role of ROS in PTER-induced lysosomal permeability and cathepsin B release, HL-60 cells were pretreated with the ROS scavenger, NAC. The immunofluorescence and Western blot results showed that NAC did not affect PTER-induced lysosomal permeability (Figure 6D) or cathepsin B release to the cytosol (Figure 6E). To further explore the significance of activated cathepsin B release in PTER-induced apoptosis, the cathepsin B-specific inhibitor, CA-074 Me, was used to suppress the effect of PTER. As shown in Figure 6F, PTER induced the same effect in the presence or absence of the cathepsin B inhibitor.

## Discussion

AML is a non-solid tumor with high mortality rates, for which novel strategies are needed to improve current treatment standards [2]. To improve clinical outcomes, identification and evaluation of novel therapeutic agents that have less toxicity in normal cells for treatment of AML are important and challenging tasks.

Anticancer mechanisms elicited by natural polyphenols have been extensively studied for many years. RESV, one of the most studied polyphenols, has powerful growth-inhibitory and apoptosis-inducing effects on cancer cells [10], [11]. Although potent anticancer effects were shown in cultured cells, potential inhibition of cancer growth by RESV in vivo is strongly limited due to its low bioavailability [12]. Therefore, it is important to consider other chemical structures, which may preserve the anticancer properties while possessing higher bioavailability.

PTER, a natural dimethylated analog of RESV with a longer half-life [16], represents an attractive option. Indeed, many in vitro and in vivo studies indicated that PTER may be a promising chemotherapeutic agent [15], [17]–[20]. In this study, we tested whether PTER treatment could be a new possibility for treating human AML and examined the mechanisms of the anticancer effect of PTER in human AML cells.

Our studies demonstrated that PTER concentration-dependently inhibited cell proliferation in five human AML cell lines, THP-1, U937, HL-60, OCI, and MV4-11. Cell proliferation is governed by the cell cycle, which is a complex and stepwise process, and uncontrolled cell proliferation is a hallmark of cancer [32]. The activity of CDKs is controlled by cyclin regulatory subunits. These form a complex with their catalytic subunit of CDKs and are regulated at a specific phase of the cell cycle [33]. In many cells, a transition through the G_1_ phase of the cell cycle and entry into the S phase require activation of cyclin/CDK complexes, mainly cyclin D/CDK6 and cyclin E/CDK2 [32]. The kinase activity of these CDK-cyclin complexes is inhibited by two classes of CKIs [34]. Members of the INK4 family (p16 INK4A and p15 INK4B) inhibit only CDK4 and CDK6, while members of the cip family (p21 Cip1 and p27 Kip1) inhibit all CDKs [35]. In HL-60 cells, PTER treatment resulted in an accumulation of cells in the G_0_/G_1_ phase of the cell cycle. PTER-induced G_0_/G_1_ cell cycle arrest was also observed in AGS human gastric cancer cells [18], in MOLT4 human lymphoblastic leukemia cells [22], and in LNCaP human androgen-responsive prostate cancer cells [19]. Moreover, treatment of HL-60 cells with PTER decreased protein levels of cyclin D3, CDK2, and CDK6 but not p15INK4B, p21 Cip1, or p27 Kip1 indicating that changes in cyclin D3, CDK2, and CDK6 protein levels seem to make a major contribution to PTER-induced G_0_/G_1_ arrest in HL-60 cells.

Apoptosis is genetically programmed cell death that plays crucial roles in both the development and maintenance of tissue homeostasis. Chemical compounds that affect apoptotic pathways and eliminate cancer cells are considered promising anticancer drugs [36]. Recently, the targeted elimination of AML cells by inducing apoptosis has emerged as a valuable strategy for combating AML [37], [38]. In this study, several hallmarks of apoptosis such as significant increases in chromatin condensation, the sub-G_1_ content, and Annexin-V-positive cells, were observed in HL-60 cells after PTER treatment. These results indicate that PTER can induce changes in phosphatidylserine externalization which belongs to apoptotic events and increases apoptotic DNA cleavage.

Mitochondria are emerging as promising targets for intervention and treatment of cancer [39]. Pan et al. [40] used transcript profiling techniques to identify cellular pathways targeted by PTER and found that it upregulated expressions of genes involved in mitochondrial functions. Caspases-8, -9, and -3 are believed to play crucial roles in mediating mitochondrion-mediated apoptosis pathways. Active caspase-8 can activate Bid, which then triggers the mitochondrial pathway to further activate caspase-9 and in turn activates the executioner, caspase-3, thus committing a cell to apoptosis [41]. Our present results showed that PTER activated the mitochondrial caspase-dependent apoptotic pathway in a concentration-dependent manner in HL-60 cells, and PTER-induced cell death was partly prevented by pretreatment with the caspase-3 inhibitor, Z-DEVE-FMK. Similar to our results, mitochondrial depolarization was also detected in PTER-treated human breast cancer cells [42] and lymphoblastic leukemia cells [22]. MAPKs are composed of several subfamilies, including ERK1/2, JNKs, and p38. These subfamilies regulate a variety of cellular responses, such as cell proliferation, differentiation, and apoptosis [43], [44]. Previous reports found that MAPKs are involved in the effects of RESV in tumor cells [45], but the role of MAPKs in PTER-induced apoptosis of tumor cells was not investigated. In this study, we further investigated activation of MAPK family proteins in PTER-treated HL-60 cells and found that only ERK and JNK were activated after PTER treatment, and the ERK-specific inhibitor, U0126, and JNK-specific inhibitor, SP600125, respectively reversed activation of caspases-8, -9, and -3 induced by PTER. Taken together, these results suggest that activation of ERK and JNK plays important roles in PTER-induced apoptosis of HL-60 cells via regulation of caspase-8, -9, and -3 activities. Moreover, PTER-induced cell death might also occur via a caspase-independent mechanism.

LMP is an alternative mechanism for inducing cell death. Depending on the lethal stimulus, the extent of LMP, the amount and type of cathepsins released into the cytoplasm, and the abundance of cathepsin inhibitors, LMP can trigger the classical MMP-caspase pathway, as well as MMP-dependent but caspase-independent apoptosis [25]. Our results showed that PTER can dramatically induce LMP and an increase of activated cathepsin B release into the cytoplasm, indicating that PTER-induced cell apoptosis might occur through an LMP-triggered MMP-caspase pathway or an LMP-triggered caspase-independent pathway. A previous report indicated that cathepsin B-dependent Bid cleavage, a prominent MMP inducer, emerged as a key connection between LMP and MMP [25]. The correlation between Bid and cathepsin B in PTER-treated leukemia cells should be further confirmed in future work. Moreover, PTER was shown to induce oxidative stress by increasing ROS levels in breast cancer cells [42], and ROS are principal inducers of LMP. ROS-dependent LMP often initiates a cell death pathway that involves sequential cathepsin translocation and MMP with cytochrome c release and caspase-dependent apoptosis [25]. In this study, we also found that PTER also can induce ROS production in HL-60 cells. To further investigate the role of ROS in PTER-induced LMP in HL-60 cells, the antioxidant, NAC, was used. Results showed that inhibition of ROS by NAC did not prevent PTER-induced LMP or cathepsin B cytosolic translocation, indicating that ROS were not involved in PTER-mediated LMP.

Several reports indicated that LMP can also initiate a caspase-independent cell death pathway. For example, microtubule-stabilizing agents such as epothilone B, discodermolide, and paclitaxel reportedly induce cathepsin B-dependent and caspase-independent cell death, as evidenced by the significant cytoprotection conferred by the cathepsin B inhibitor, CA074-Me (but not the broad-spectrum caspase inhibitor, Z-VAD-FMK) [46]. Because cathepsin B is one of the most abundant lysosomal proteases released from lysosome to induce cell death after LMP [25]. Moreover, cathepsin B has also been implicated as an inducer of LMP. For example, hepatocytes from cathepsin B^−/−^ mice display less LMP after exposure to TNF-α, which may reflect the direct induction of LMP by cathepsin B [47]. According to these observations, an amplification loop may exist through LMP induces cathepsin B activation, and cathepsin B then further triggers LMP. In our study, we used CA074-Me, the cathepsin B-specific inhibitor, as the blocker of LMP-mediated cell death. Surprisingly, pretreatment of HL-60 cells with CA074-Me did not prevent cell death induced by PTER in our study. This might have been because the cytotoxic effect induced by the release of lysosomal hydrolases not only involves cathepsin B but also includes other cathepsins (e.g., cathepsin D, E, L, and H), and only blocking the activity of cathepsin B cannot sufficiently reverse the toxic effect of PTER. A previous report indicated that downregulation of heat shock protein (HSP) 70, which protects lysosomal membranes from LMP-inducing stimuli, induces caspase-independent cell death in breast cancer cells [48]. Moreover, PTER has also been reported to induce LMP-mediated cell death which depends on HSP70 levels in several solid tumor cell lines. This report indicated that endogenous HSP70 levels were found to be higher in cells with lower PTER susceptibility (HT29 and MCF-7 cells) than cells with higher PTER susceptibility (A375 and A549 cells) and knockdown of HSP70 in HT29 or MCF-7 cells can increase the PTER-induced cell death [49]. However, in our preliminary study, the endogenous HSP70 levels did not correlate with their susceptibility for PTER-mediated cell death in AML cell lines (Figure S6 in File S1). In addition to HSP70, the apoptosis-inducing factor (AIF) was shown to be an important effector of caspase-independent cell death induced by LMP [25]. The exact roles of HSP70 and AIF in PTER-induced apoptosis of leukemic cells must be further investigated in future work.

**Figure 7:**
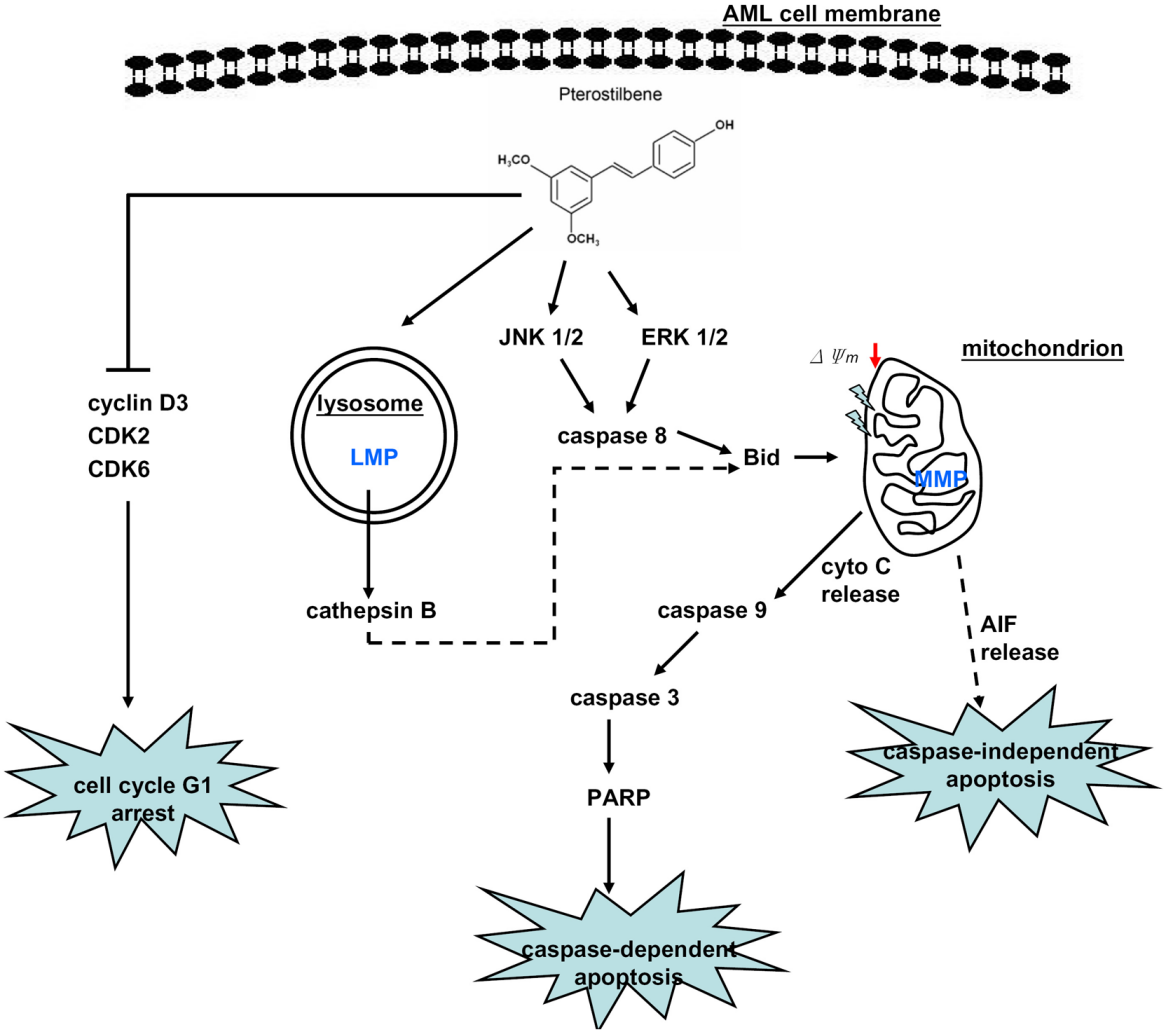
Proposed signal transduction pathways by which pterostilbene (PTER) inhibits the growth of HL-60 cells. ‘Bold solid lines’ indicate pathways affected by PTER. ‘Bold dashed lines’ indicate hypothetical pathways which might be affected by PTER. LMP, lysosomal membrane permeabilization; MMP, mitochondrial membrane permeabilization; cyto C, cytochrome C.

In summary, the present study demonstrated that PTER possesses an antileukemic effect on AML cells, and its anticancer activity was attributed to its induction of cell cycle arrest and apoptosis. The schematic mechanism is illustrated in Figure 7 and indicates that AML cell apoptosis elicited by PTER is mediated through MAPKs/caspases/MMP-dependent pathway, the disruption of LMP might also play a role in PTER-mediated apoptosis. The cell cycle arrest induced by PTER is mediated through downregulation of cyclin D3, CDK2 and CDK6 expressions. Our findings have elucidated the underlying mechanisms of the antileukemic effects of PTER and revealed that PTER may be a useful candidate as a chemotherapeutic agent for AML therapy.

## Supporting Information

File S1 Figure S1, Effect of pterostilbene on U937 cell apoptosis as well as caspases activation. Figure S2, Effect of pterostilbene (PTER) on mitochondrial membrane permeability. Figure S3, Effects of ERK and JNK specific inhibitors on pterostilbene-induced activation of ERK and JNK. Figure S4, Effect of JNK specific inhibitor, JNK-IN-8 on pterostilbene-induced activation of caspases. Figure S5, Effect of pterostilbene on the ERK and JNK activation. Figure S6, The endogenous HSP70 levels in five AML cells.
(DOCX)
