# Supplementary material for: Pterostilbene Simultaneously Induced G0/G1-Phase Arrest and MAPK-Mediated Mitochondrial-Derived Apoptosis in Human Acute Myeloid Leukemia Cell Lines
Source: PLoS One. 2014 Aug 21;9(8):e105342. doi: 10.1371/journal.pone.0105342 (PMC4140770; doi:10.1371/journal.pone.0105342)
Supplement: File S1 — Figure S1, Effect of pterostilbene on U937 cell apoptosis as well as caspases activation. Figure S2, Effect of pterostilbene (PTER) on mitochondrial membrane permeability. Figure S3, Effects of ERK and JNK specific inhibitors on pterostilbene-induced activation of ERK and JNK. Figure S4, Effect of JNK specific inhibitor, JNK-IN-8 on pterostilbene-induced activation of caspases. Figure S5, Effect of pterostilbene on the ERK and JNK activation. Figure S6, The endogenous HSP70 levels in five AML cells. (DOCX) [file pone.0105342.s001.docx]

**Figure S1**

**A B
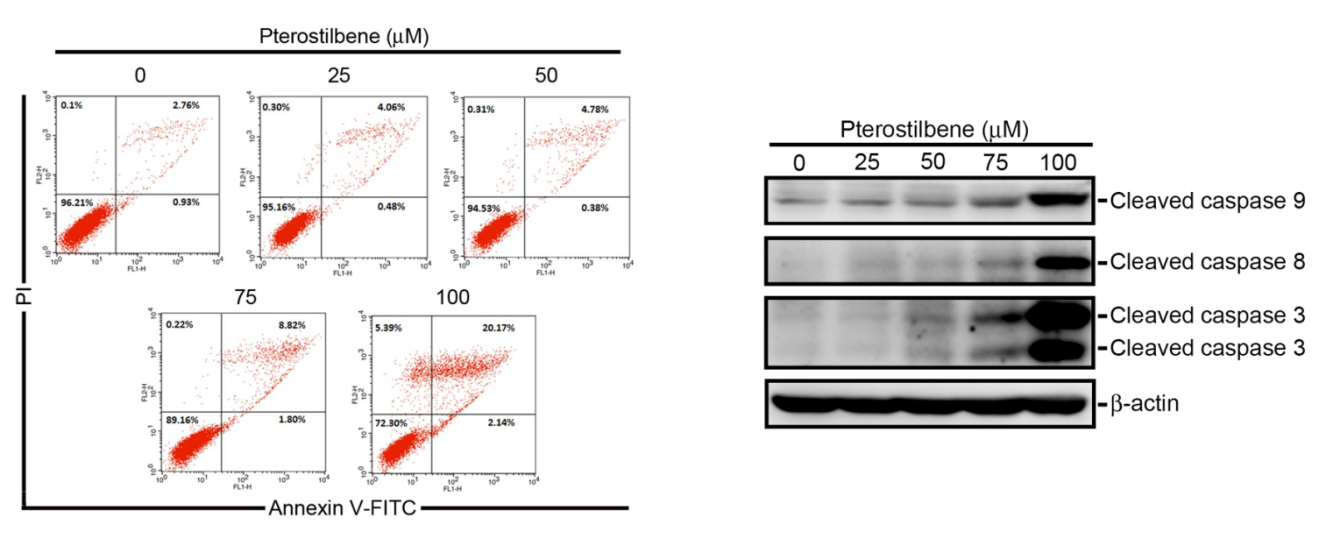
**

**Figure S1. Effect of pterostilbene on U937 cell apoptosis as well as caspases activation.** (A) Analysis of cell apoptosis by Annexin-V and propidium iodide (PI) double-staining flow cytometry after treatment with different concentrations of pterostilbene (0~100 µM) for 24 h. (B) Expression levels of cleaved caspases-3, -8, and -9 were assessed by a Western blot analysis after treatment with various concentrations of pterostilbene (0~100 µM) for 24 h.

**Figure S2**

**
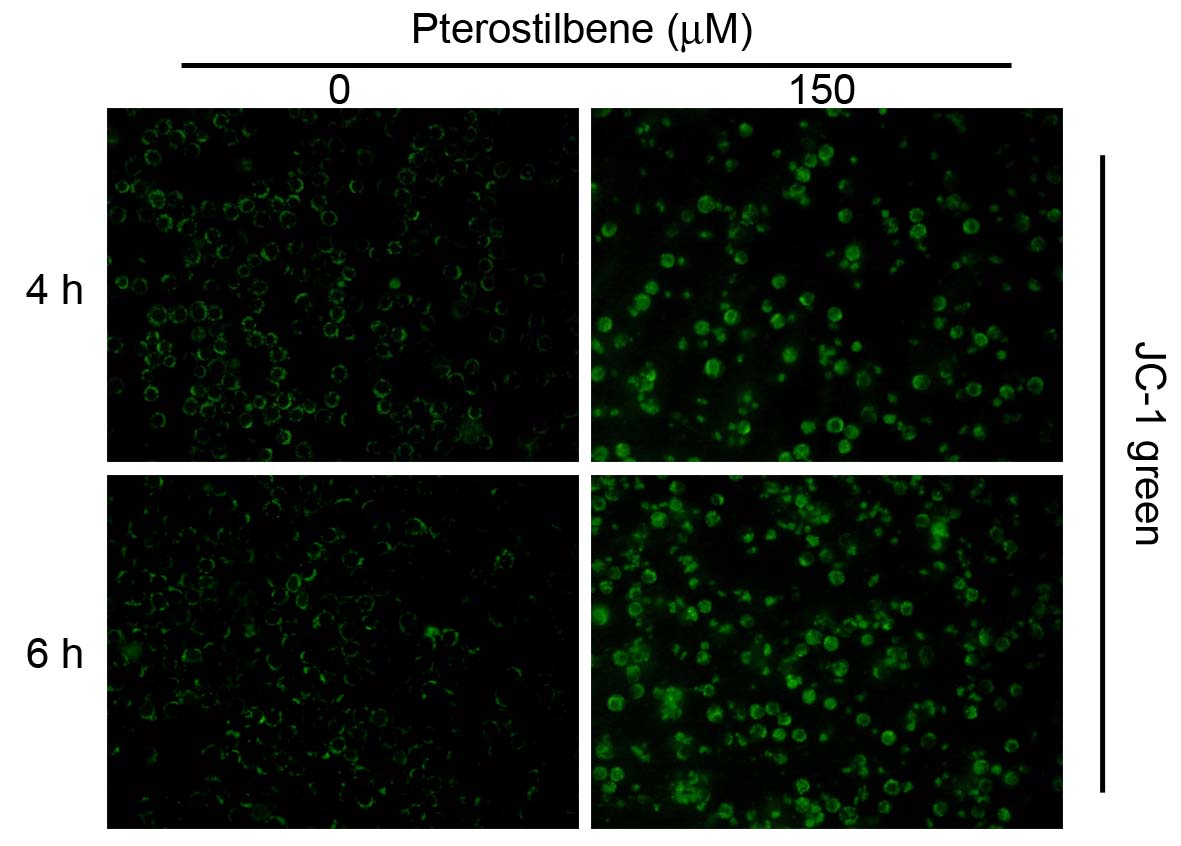
**

**Figure S2. Effect of pterostilbene (PTER) on mitochondrial membrane permeability.** Immunofluorescence analysis showed that green-fluorescent monomeric form increases in HL-60 cells after treatment with 150 μM PTER for 4 or 6 h. Original magnification, 200X.

**Figure S3**

**
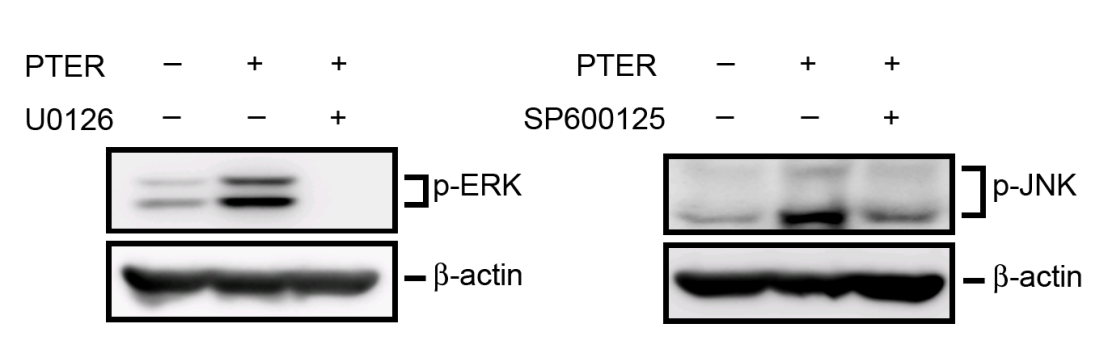
**

**Figure S3. Effects of ERK and JNK specific inhibitors on pterostilbene-induced activation of ERK and JNK.** HL-60 cells were pretreated with 20 µM U0126 (an ERK inhibitor) or SP600125 (a JNK inhibitor) for 1 h, treated with 100 µM PTER for another 24 h, and then the phosphorylation levels of ERK1/2 and JNK1/2 were assessed by a Western blot analysis.

**Figure S4**


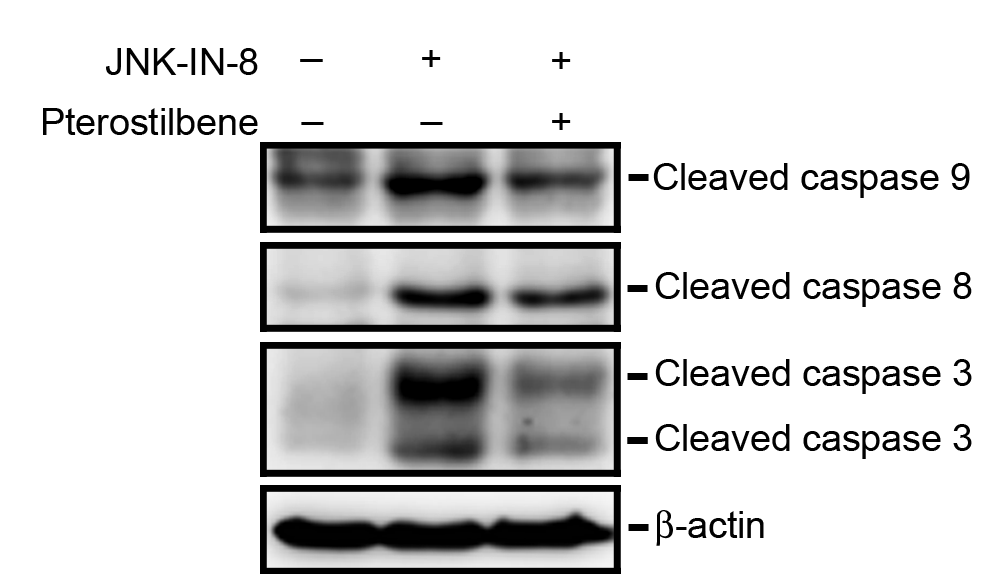


**Figure S4. Effect of JNK specific inhibitor, JNK-IN-8 on pterostilbene-induced activation of caspases.** HL-60 cells were pretreated with or without 1μM JNK-IN-8 for 1 h followed by pterostilbene (100 μM) treatment for an additional 24 h. Expression levels of cleaved caspase-3, -8, and -9 were determined by a Western blot analysis.

**Figure S5**

**
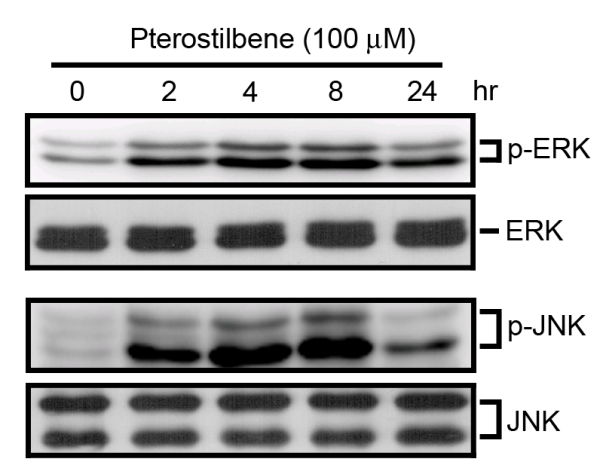
**

**Figure S5. Effect of pterostilbene on the ERK and JNK activation.** Phosphorylation levels of ERK1/2 and JNK1/2 were assessed by a Western blot analysis after treatment with pterostilbene (100 μM) for different time points (0-24 h).

**Figure S6**

**
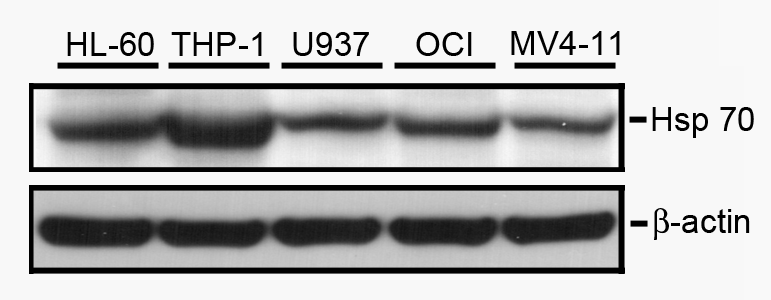
**

**Figure S6. The endogenous HSP70 levels in five AML cells**. Endogenous levels of HSP70 in five AML cell lines were assessed by a Western blot analysis.
